# Penetrating Abdominal Trauma With Small Bowel Evisceration: A Clinical Image

**DOI:** 10.1002/ccr3.72519

**Published:** 2026-04-10

**Authors:** Chukwuka Elendu

**Affiliations:** ^1^ Federal University Teaching Hospital Owerri Nigeria

**Keywords:** bowel evisceration, damage‐control trauma care, interpersonal violence, penetrating abdominal trauma, surgical emergency

## Abstract

Penetrating abdominal trauma with visible bowel evisceration signals severe underlying injury and mandates immediate surgical consultation. Early recognition, careful handling of exposed viscera, and avoidance of inappropriate bedside manipulation are critical to prevent ischemic injury, infection, and adverse outcomes while definitive operative care is arranged.

1

A young adult male presented following a penetrating abdominal injury sustained during an interpersonal assault. On physical examination, he was conscious but in distress, with visible abdominal wall disruption, active external bleeding, and extrusion of edematous small bowel loops through the left anterolateral abdominal wall defect, consistent with traumatic abdominal evisceration (Figures 1, 2, 3). The exposed bowel showed active oozing, with no immediate evidence of gross contamination by foreign material.

**FIGURE 1:**
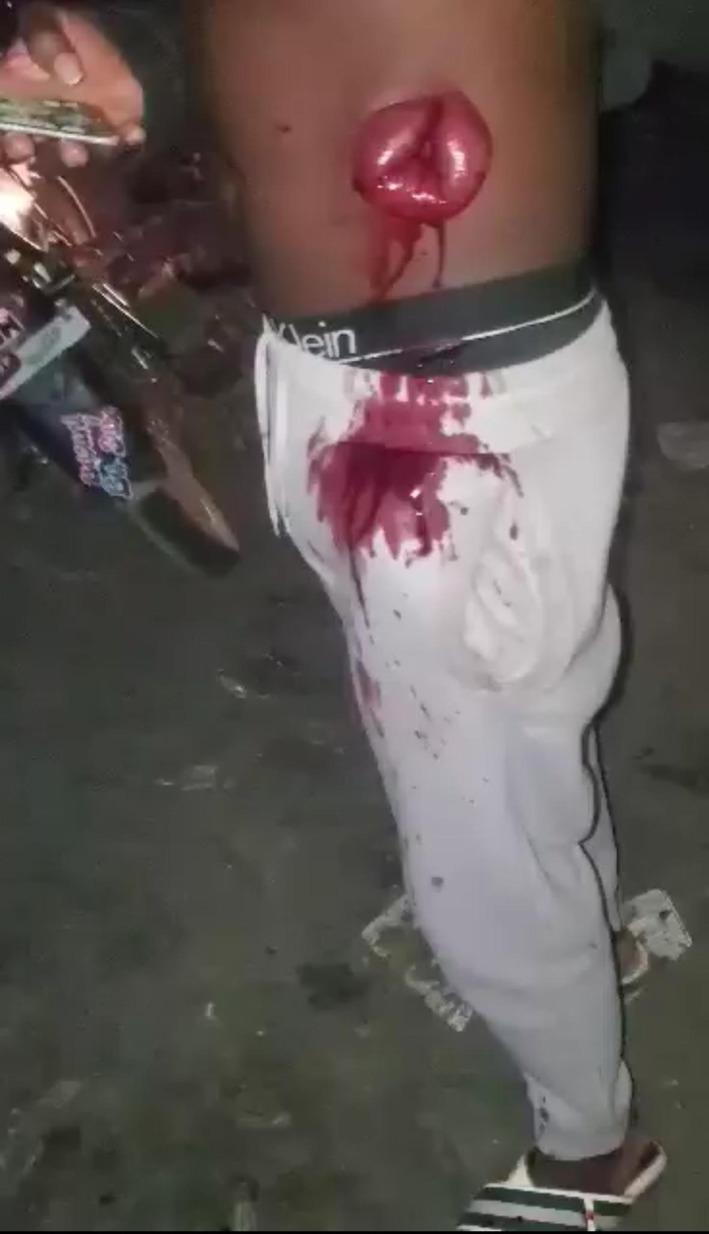
Clinical photograph showing penetrating left anterolateral abdominal wall trauma with extrusion of small bowel loops through a full‐thickness abdominal wall defect, consistent with traumatic abdominal evisceration. Active external bleeding is visible at presentation.

**FIGURE 2:**
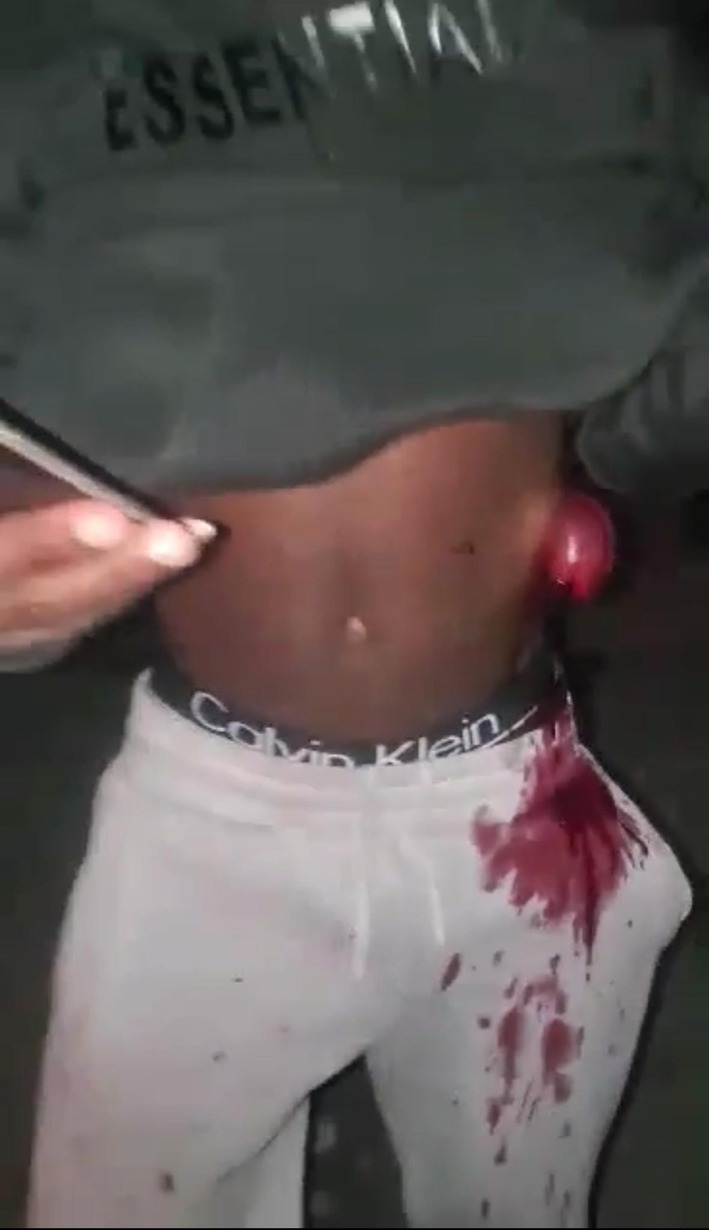
Alternate clinical view highlighting the relationship between the eviscerated bowel and the abdominal wall defect, emphasizing the extent of visceral exposure prior to operative management.

**FIGURE 3:**
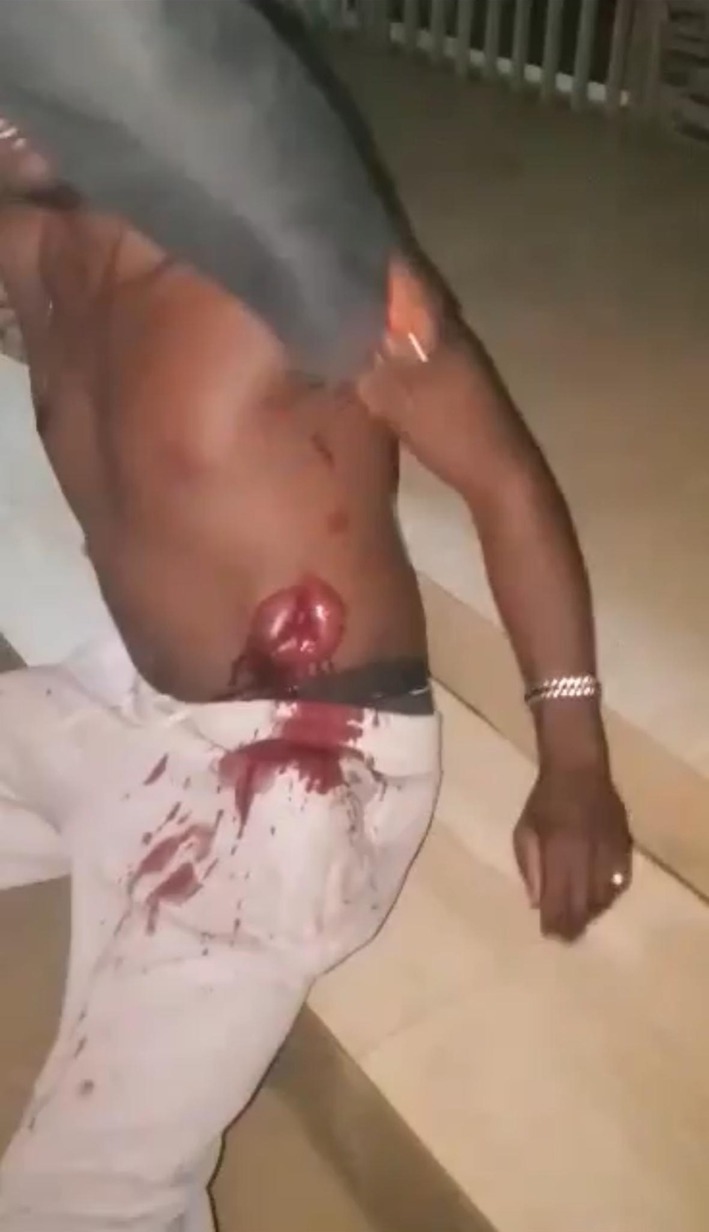
Wider clinical view demonstrating the overall extent of small bowel evisceration and surrounding abdominal wall disruption, providing anatomical context for the injury.

The eviscerated bowel was covered with sterile saline‐soaked dressings to prevent desiccation and thermal injury, in keeping with established trauma care principles. The patient was positioned supine with minimal manipulation of the protruding bowel, and preparations were made for urgent transfer for definitive surgical management.

Following stabilization, the patient underwent emergent exploratory laparotomy, which revealed small bowel injury without extensive contamination. Surgical intervention was performed accordingly, with successful management of the injured bowel. The patient had a favorable immediate postoperative course and remained clinically stable in the early postoperative period.

## 2 Discussion

This clinical image highlights the critical recognition and management of penetrating abdominal trauma complicated by small bowel evisceration, a finding that reliably indicates full‐thickness abdominal wall violation and a high probability of underlying visceral injury [1]. In contrast to blunt abdominal trauma, evisceration following penetrating injury represents a strong operative indication, as clinical examination and imaging alone may fail to detect occult bowel or mesenteric injury.

Preoperative manipulation of eviscerated bowel—such as aggressive irrigation or attempted reduction—should be avoided, as this may exacerbate contamination, compromise mesenteric perfusion, or mask evolving ischemia [2]. Instead, temporary coverage with sterile saline‐moistened dressings preserves bowel viability while minimizing iatrogenic injury prior to operative exploration.

From a pathophysiological standpoint, bowel evisceration frequently coexists with mesenteric disruption and microvascular compromise that may not be immediately apparent, even in hemodynamically stable patients. Such occult injury increases the risk of delayed perforation, intra‐abdominal contamination, and subsequent sepsis if intervention is delayed [1, 3].

In the present case, early surgical intervention allowed definitive management of the bowel injury and resulted in a favorable immediate postoperative outcome, underscoring the benefit of timely operative decision‐making. Collectively, this image reinforces key learning points: bowel evisceration in penetrating trauma mandates urgent surgical evaluation, careful preoperative handling to prevent secondary injury, and timely operative management to optimize outcomes.

## Author Contributions

**Chukwuka Elendu:** conceptualization, data curation, formal analysis, investigation, methodology, project administration, supervision, validation, visualization, writing – original draft, writing – review and editing.

## Funding

The author has nothing to report.

## Disclosure

The views expressed in this report are solely those of the author and do not represent the official positions of any affiliated institutions.

## Ethics Statement

The author has nothing to report.

## Consent

Written informed consent was obtained from the patient for publication of this case image and accompanying clinical data. All identifying information has been anonymized to protect patient privacy.

## Conflicts of Interest

The author declares no conflicts of interest.

## Data Availability

No datasets were generated or analyzed during the current study, and all relevant clinical information is included within the article.
